# Physical activity, time use, and food intakes of rural households in Ghana, India, and Nepal

**DOI:** 10.1038/s41597-020-0414-x

**Published:** 2020-03-03

**Authors:** Giacomo Zanello, C. S. Srinivasan, Fiorella Picchioni, Patrick Webb, Paul Nkegbe, Radhika Cherukuri, Shailes Neupane

**Affiliations:** 1 University of Reading, Reading, United Kingdom; 2 Natural Resources Institute, University of Greenwich, Kent, United Kingdom; 3 Tufts University, Boston, United States of America; 4 University for Development Studies, Tamale, Ghana; 5 National Institute of Rural Development and Panchayati Raj, Hyderabad, India; 6 Valley Research Group, Lalitpur, Nepal

**Keywords:** Agriculture, Nutrition, Developing world

## Abstract

With more than 820 million undernourished people living in rural areas of low- and middle-income countries (LMICs), ending hunger and ensuring access to food by all is a global priority. In the past few decades, the adoption of technological innovations in the agricultural sector and related crop yield improvements have not led to expected improvements in the nutritional status of rural households in many LMICs. The increased energy expenditure associated with the adoption of productivity-enhancing innovations may provide an important explanation of the disconnect between agricultural productivity enhancements and improved nutritional outcomes. We develop a methodology for generating reliable livelihood energy/calorie expenditure profiles for rural agricultural households using research-grade accelerometer devices. We integrate the data on physical activity and energy expenditure in rural households with data on time-use and food intakes to generate a data set that provides a unique window into rural livelihoods. This can be a valuable resource to analyse agriculture-nutrition impact pathways and improve the welfare of rural and agricultural households.

## Abstract (summary)

**Table:** 

Measurement(s)	physical activity
Technology Type(s)	Accelerometer • Survey
Factor Type(s)	geographic location • community type
Sample Characteristic - Environment	rural area
Sample Characteristic - Location	Ghana • Nepal • India

Machine-accessible metadata file describing the reported data: 10.6084/m9.figshare.11871537

## Background & Summary

Undernutrition remains a significant challenge in rural areas of low- and middle income countries (LIMCs), with more than 820 million people in the world not meeting the minimum caloric requirements in 2018^1^. Effective and immediate coordinated actions are needed to meet the Sustainable Development Goals (SDGs) of ending hunger and ensuring access to food by all (SDG 2.1) and of eliminating all forms of malnutrition (SDG 2.2) by 2030. Notwithstanding the adoption of technologies aimed at increasing yields, in many developing countries there appears to be a perplexing disconnect between agricultural productivity growth and expected improvements in nutrition status^2,3^.

Productivity-enhancing agricultural interventions impact the calorie deficits of the undernourished via their effects on energy intakes and energy expenditure. At the level of the individual, these effects are further mediated by intra-household allocations of consumption and labour. Intra-household allocations of time, labour and consumption have been recognised as significant determinants of the adoption of productivity-enhancing agricultural innovations and their impact on the nutritional status of individual household members^4,5^. Gender and age-related differentiation in intra-household allocation decisions have been observed to have an important role in explaining the nutrition impacts of productivity-enhancing innovations in developing country contexts^6^. A key impact pathway to nutrition is the effect of innovations on energy expenditure and energy intakes of individuals^7^, mediated by intra-household allocation decisions. In fact, nutritional outcomes of productivity-enhancing agricultural interventions are determined by the changes in energy intakes facilitated by productivity enhancements – such as mechanization or adoption of new technology – as well as by the changes in energy expenditures called for by the intervention for different members of the rural household. While the gains in consumption for an individual from an intervention are mediated by the technology and adoption efficiency parameters influencing the gains in production, market participation and income utilisation decisions and intra-household food allocation patterns, the energy expenditure associated with the intervention will depend on the physical activity demand created by it and the intra-household allocation of labour. However, reliable and accurate empirical measurement of energy expenditure impacts of agricultural productivity-enhancing innovations at the level of the individual remains challenging.

Scientific (physiological) empirical measurement of energy expenditure profiles in developing countries (e.g., using the “gold-standard” method of Doubly Labelled Water (DLW) and indirect calorimetry methods) has been hampered by the high cost and difficulty in applying standard methods and protocols in field settings among free-living populations^8,9^. Recent advances in technology allow the use of wearable of accelerometry devices to capture energy expenditures. Energy expenditure data from accelerometry devices have been validated against data from DLW and heart monitors in free-living population^10^, yet their use in agriculture settings has been limited so far^11,12^. Accelerometers offer an unobtrusive tool for obtaining energy expenditure data in free-living populations. The dataset described in this paper was generated by a project which aimed to develop robust energy expenditure profiles of rural households in LMICs taking advantage of wearable research-grade accelerometers and integrate energy expenditure data with data on time-use and food intakes.

Together with data on energy expenditure from accelerometry devices, data was also collected on household and individual characteristics and daily individual level time-use and food intakes. The data collection took place from June 2017 to November 2018 in rural areas of Ghana, India, and Nepal. The selected areas represent diverse agroecological zones and agricultural practices. In each country, we sampled 20 households and invited the head of the household and the spouse to participate in the study by wearing an accelerometry device for four full non-consecutive weeks across the agricultural season. These correspond to four key phases of the agricultural cycle, from land preparation to ploughing, land maintenance, and harvest.

The final published dataset^13^ includes household and individual characteristics and raw accelerometer data (Table 1). This unique resource has the potential to generate an integrated data sets that links physical activity with time use and food intake. Empirical analysis can facilitate a clearer delineation of the agriculture-nutrition impact pathways and improve the welfare of rural and agricultural households. Specifically, the data can be used to:

- Better estimate the incidence, depth and severity of undernutrition and poverty, through the lens of energy intake-energy expenditure balance.
- Better understand intra-household labour and physical activity allocation decisions, including those associated with the adoption of productivity-enhancing agricultural innovations.
- Better understand the link between productivity-enhancing interventions and nutrition outcomes for individuals within a household.

**Table 1:** Dataset summary.

Sample	Location	Selection criteria and size
Household survey	Ghana, India, Nepal	Random sample of 60 households stratified by land endowments (20 in each country). In each country 10 households were from a community practising rain-fed agriculture, while the other 10 had irrigation facilities.
Individual survey	Ghana, India, Nepal	Daily individual survey of the household head and the spouse collecting information on time use and food intakes. Survey spanning four non-consecutive weeks during the 2017–2018 agricultural season. Full sample of 120 individuals resulting in 3,360 days/person (40 individuals in each country and 1,120 days/person). The time-use data yielded data for 80,640 hours (26,880 hours in each country)
Accelerometry data	Ghana, India, Nepal	Accelerometry data capturing individual movements across three axes (X, Y, Z) matching the individual survey (four non-consecutive weeks during the 2017–2018 agricultural season) stored at 1 sec/interval for each individual.

The detailed examination of energy expenditure, time use patterns and food intake can facilitate the development of approaches to nutrition and welfare enhancement that are not focused on productivity/yield improving innovations alone. Considering time and energy trade-offs between a wider range of different rural livelihood activities in policy design can help to tailor policies specific to different needs of women and men in rural areas.

## Methods

### Sampling

**Fig. 1:**
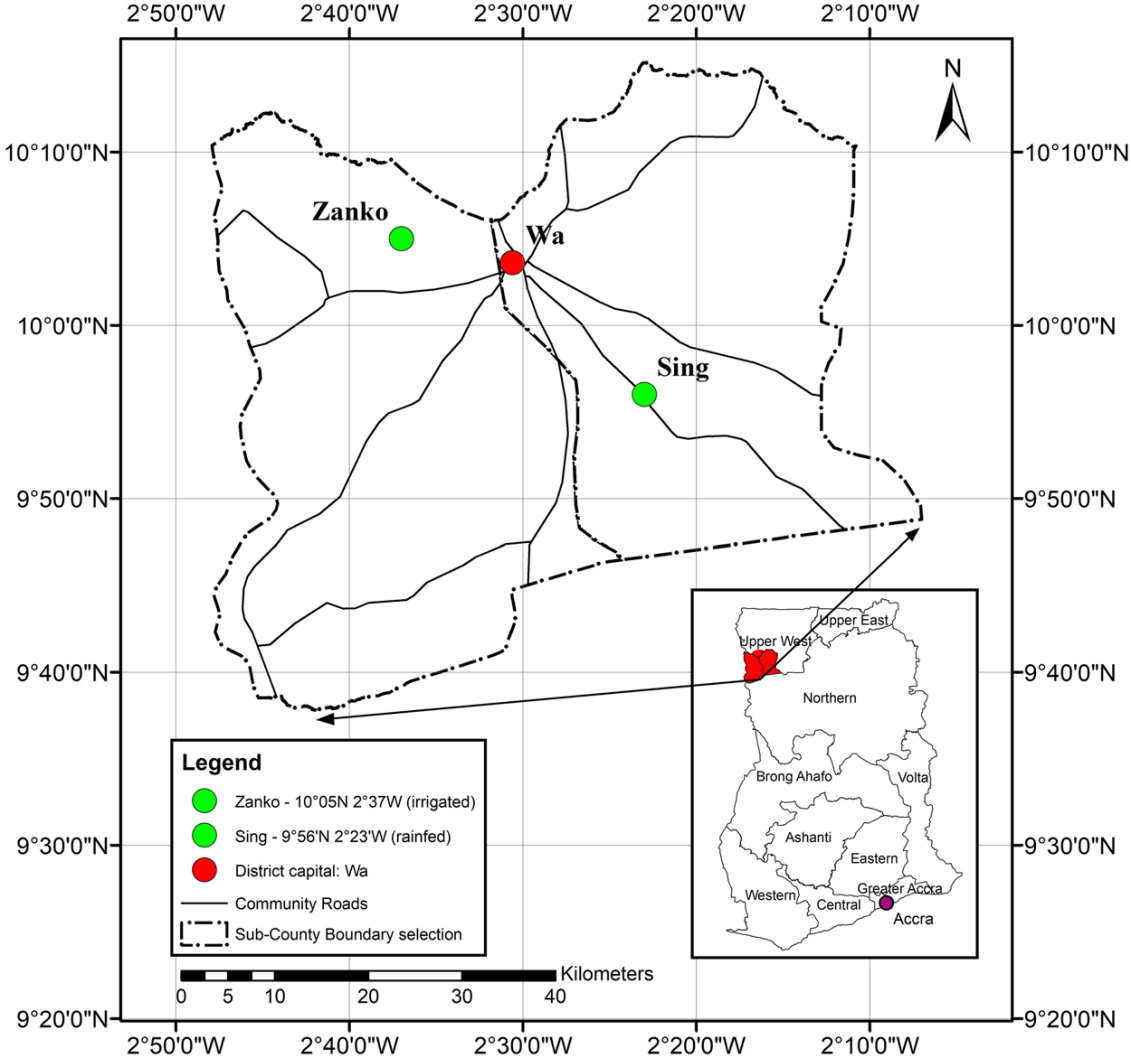
Field sites in Ghana.

**Fig. 2:**
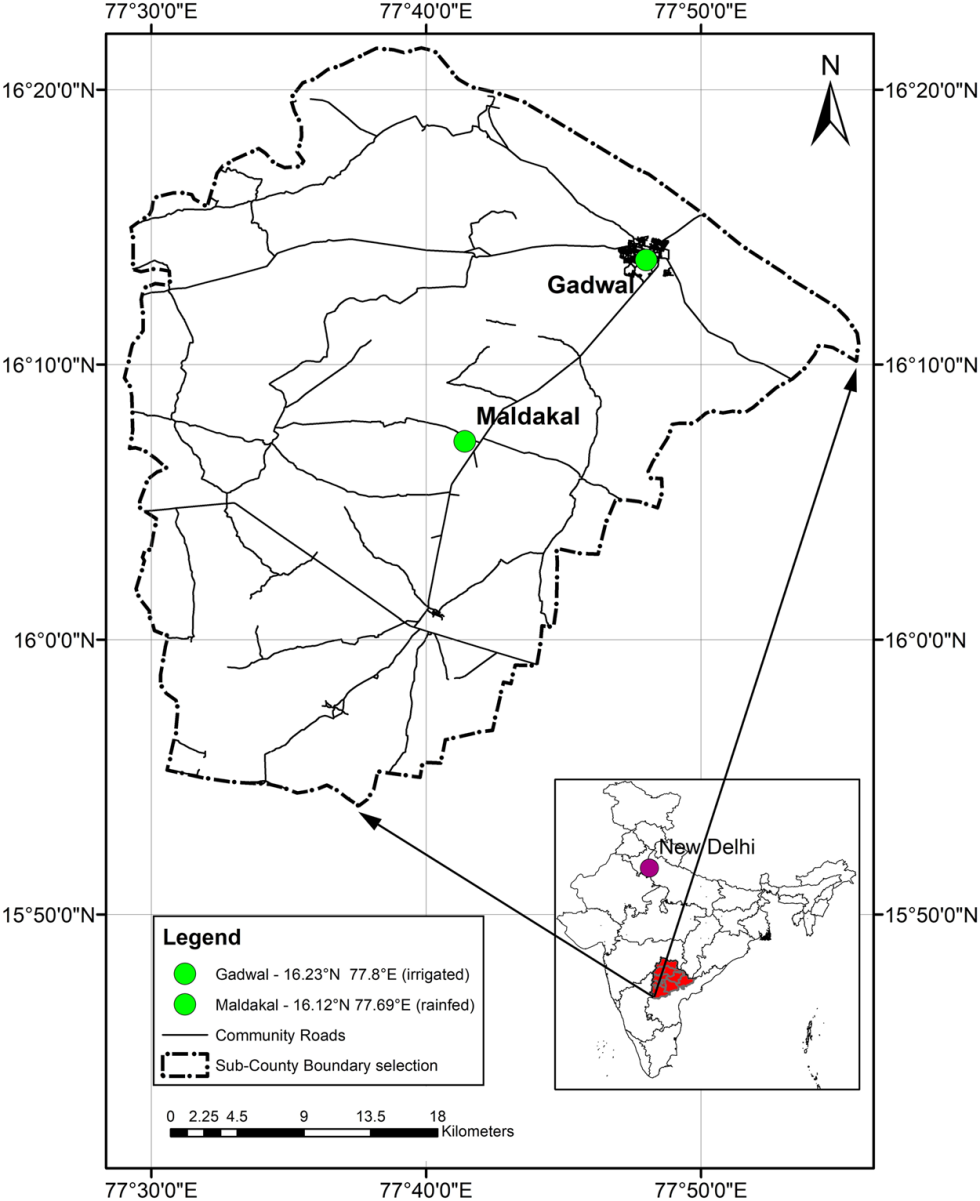
Field sites in India.

**Fig. 3:**
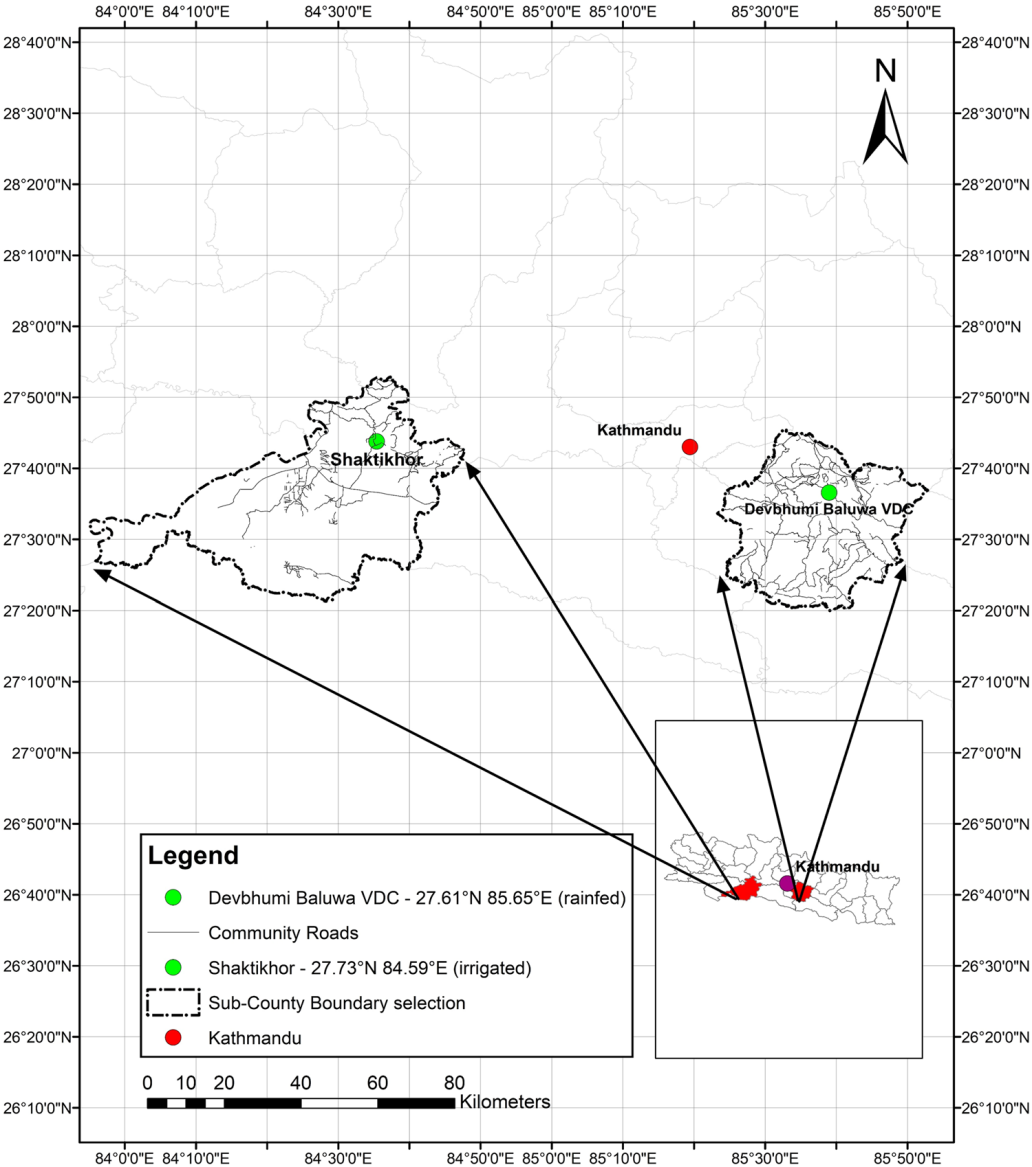
Field sites in Nepal.

Data were collected in two communities – one adopting rainfed agriculture and one with irrigation infrastructure–in rural areas of Ghana, India, and Nepal (Figs. 1–3). The sample of households was split across the two agricultural systems to capture the different patterns of time-use and physical activity prevalent in irrigated and non-irrigated households. Irrigation facilities can release time in some agricultural activities, such as watering (a duty traditionally performed by women)^14^, and may be associated with a higher level of mechanisation. The stratification of the sample households by landholding size (see below) was designed to control for differences in socio-economic characteristics and endowments across the two systems. In Ghana fieldwork took place in the Upper-West region (June 2017–May 2018), in India in Jogulamba Gadwal District in Telangana State (June–December 2018), and in Nepal in Province n. 3 (June 2017–September 2018).

A few weeks before the commencement of data collection, communities were visited to make initial contacts and to build rapport with community leaders. A detailed briefing on the research was also provided. A local member of the field team compiled a listing of households in the community together with the land size. During a second visit, which took place between a week to three days prior to the survey, the list was used to draw a random sample of households to participate in the survey based on the size of the land being cultivated (smallholders, medium and large-holders). The classification of land endowments varies across countries based on local context. In Ghana, the Ministry of Food and Agriculture defines smallholders as those cultivating less than 2.5 hectares of land, medium 2.5–5.0 ha, large holders >5 ha. In India, based on the Agriculture Census (2010–11) the Ministry of Agriculture & Farmers Welfare defines smallholders (<2 ha), medium (2–4 ha), and large farmers (>5 ha). In Nepal, the High-Level Commission on Scientific Land Reform (2010) categorises 0.1–0.5 ha as small, 0.5–3 ha as medium and >3 ha as large. Table 2 reports the sample allocation.

**Table 2:** Sample allocation (stratified by land endowment).

Size	Ghana	India	Nepal
Smallholders	36%	35%	22%
Medium	41%	35%	74%
Larger-holders	23%	30%	4%
Average size (ha)	4.52	4.05	1.52

In each community, we therefore sampled 10 households (20 households in each country for both rain-fed and irrigation conditions). In the randomly selected households, we invited the head of the household and the spouse to participate in the study. To be part of the study, the respondents had to be currently economically active and aged between 16–64 years.

### Protocol and survey instruments

To capture different rural activities throughout the agricultural season, we invited each participant to wear the accelerometry device for four non-consecutive weeks across the agricultural season. These were scheduled to match four key agricultural phases: (1) land preparation, (2) sowing and seeding, (3) maintenance (e.g., activities related to weeding and fertilizing), and (4) harvest. The accelerometry literature recommends 4–7 days of wear^15^ to mitigate potential non-wear time and avoid participants fatigue. The timing of each phase in each community is reported in Table 3.

**Table 3:** Fieldwork timing.

			2017								2018											
			5	6	7	8	9	10	11	12	1	2	3	4	5	6	7	8	9	10	11	12
Ghana	Rainfed	Land preparation (Week 1)	X																			
		Seeding and sowing (Week 2)	X	X																		
		Land maintenance (Week 3)			X																	
		Harvest (Week 4)					X	X														
	Irrigated	Land preparation (Week 1)							X	X	X											
		Seeding and sowing (Week 2)								X	X											
		Land maintenance (Week 3)									X	X										
		Harvest (Week 4)											X									
Nepal	Rainfed	Land preparation (Week 1)											X	X								
		Seeding and sowing (Week 2)													X							
		Land maintenance (Week 3)														X						
		Harvest (Week 4)																	X			
	Irrigated	Land preparation (Week 1)		X																		
		Seeding and sowing (Week 2)			X																	
		Land maintenance (Week 3)				X																
		Harvest (Week 4)					X															
India	Rainfed	Land preparation (Week 1)															X					
		Seeding and sowing (Week 2)															X					
		Land maintenance (Week 3)																	X			
		Harvest (Week 4)																			X	
	Irrigated	Land preparation (Week 1)														X	X					
		Seeding and sowing (Week 2)														X	X					
		Land maintenance (Week 3)																	X			
		Harvest (Week 4)																			X	

The accelerometry data were recorded with ActiGraph^TM^ GT3X+ devices, a research-graded accelerometer. The device is as small as a wristwatch, rugged, and water resistant, and suitable for 24-hour of continuous use. It is inserted in an elastic belt and worn around the waist, under or over clothing. The lack of a screen or any on/off switch prevents users from interfering with the devices or from altering their activity in response to device feedback. These characteristics make it suitable for research in LMIC settings^11^. The accelerometer data were collected at 30 Hz frequency and recorded at 1-second intervals (mean).

A series of surveys complemented the data from accelerometry devices (Table 4):

- *Household questionnaire:* An initial household questionnaire was administered to the head of the household on the first day of the data collection. The survey captured a wide range of household level information, including household composition, dwelling characteristics, asset ownership (to generate a wealth index), employment and labour force activities, land and agriculture, livestock, decision-making in the household, and access to infrastructure.
- *Individual questionnaire:* An individual questionnaire was administered daily to each participant and the nature of the information collected varied depending on the time of the data collection. At the beginning of the data collection we collected self-reported health information. Anthropometrics information (weight and height) of the participants were collected on the first day of Week 1 and Week 3. Every day a 24-hour recall module was administered to collect information from the individual participants about the type and amount of food and beverages they consumed at each meal (breakfast, mid-morning, noon, mid-afternoon, evening, before going to bed) in the previous 24-hour period. From the second day of the week a time-use questionnaire was administrated to record in 1-hour time slots primary and secondary activities (if any). Each participant was freely reporting their activities, providing more granular details of the activities. Time use recall was limited to the timeframe 5 am to 10 pm in India and 4 am to 10 pm in Ghana and Nepal (we assumed participants were resting/sleeping outside these time frames). On the last day of each week an extended time-use module was administered aimed at capturing more in-depth information on the labour allocation in agricultural activities performed during the week.

**Table 4:** Survey instruments.

	Week 1							Week 2							Week 3							Week 4						
	1	2	3	4	5	6	7	1	2	3	4	5	6	7	1	2	3	4	5	6	7	1	2	3	4	5	6	7
Accelerometer data	X	X	X	X	X	X	X	X	X	X	X	X	X	X	X	X	X	X	X	X	X	X	X	X	X	X	X	X
Household data	X																											
Anthropometrics data	X														X													
Self-reported health data	X																											
24-hours food intakes	X	X	X	X	X	X	X	X	X	X	X	X	X	X	X	X	X	X	X	X	X	X	X	X	X	X	X	X
24-hours time-use		X	X	X	X	X	X		X	X	X	X	X	X		X	X	X	X	X	X		X	X	X	X	X	X
Extended time-use							X							X							X							X

### Households replacement protocol

In the event individuals (or households) surveyed during the previous week were not available subsequently, we designed and followed a protocol to replace the participant(s). If the male or female respondents within a household were unwilling or unable to take part to the data collection (having participated in the previous week), enumerators were instructed to first find a replacement within the household that matched the gender and inclusion criterion(economically active and between 16–64 years old). If this was not possible, a replacement household was randomly selected within the same strata (land size) from the initial listing of households in the community. The new household was then surveyed and a household questionnaire administered, followed by the individual questionnaires in that week and subsequent weeks.

## Ethical Approval

The research and study design were reviewed and approved by the Ethics Committee at the University of Reading (#00460D) and by the relevant in-country research ethics committees. Informed consent was obtained from all subjects involved in the research. The consent form did not explicitly inform the respondents that anonymised data could be stored publicly and shared through an approved platform by the funder (UK DFID). However, it may be noted that data was collected from communities with a large number of households (100–2500). The geographic co-ordinates of the field sites provided in the data set cover a five-mile radius which covers neighbouring communities from which no data was collected. After careful consideration of the anonymised data archived with the UK Data Archive^16^, we are confident that appropriate steps have been taken to prevent “reverse” identification of participants minimising the risks of violation of anonymity.

## Data Records

The datasets and questionnaires (including participant information sheet and consent form) are archived in UK Data Archive^13^ (10.5255/UKDA-SN-853777) in zipped Stata and PDF format, respectively. Datasets have been anonymised throughout, identifiers removed, and time of accelerometers set to 1 January 1960. There are two sets of data:

1. **Households and individual characteristics:** Country level datasets provide information related to the household. The unit of observation is the household. Individual characteristics (self-reported health status), and time use and food consumption data are reported at individual-day level. The translation of the activities is provided in a Excel file. The files are included in the Survey_Data.zip.
2. **Accelerometry:** Accelerometry data was originally collected at 30 Hz and in order to reduce the file size the raw data was pre-processed to take an average of the movements across the three axes (X, Y, Z) at one-second level. The resulting individual level accelerometry data have been archived in individual zipped files at country level. The file naming follows the following template COUNTRY_HH_IND_WEEK, e.g. GH_HH1_M_W1 contains the accelerometry data of the Male (M) participant of the Household 1 (HH1) during land preparation (W1) in Ghana (GH). The accelerometer data is at one-second level.

## Technical Validation

The data collection protocols were developed from two pilot projects in Ghana^11^ and India. The field-experience we gained was instrumental in the design and management of the fieldwork, considering ethical implications of collecting accelerometer measurement in rural populations^17^.

### Sample attrition

In India, no replacements were needed throughout the data collection. In Ghana, in the rainfed community, two households moved out of the village before the start of planting season (Week 2) and were replaced for the remainder of the study. In the rainfed community in Nepal, a food poisoning outbreak hospitalised six households just before the harvest (Week 4). Given the extent of the outbreak in the community, the survey team was able to find a replacement only for three households. The short harvest season did not allow for an extension of the fieldwork and replacement of the remaining households. Therefore, we ended up with an unbalanced panel of 22 and 23 households in Ghana and Nepal, respectively.

### Enumerator teams

In each country, enumerators were selected by local partners based on previous experience of administering household questionnaires, relevant education (BSc or MSc in social science or agriculture), and knowledge of the local language. In most cases, enumerators were originally from the same District or Province of the selected field sites. Enumerators were coordinated by a Field Coordinator. Before the start of the data collection, enumerators were trained for a minimum of three days. Sessions covered the content of the questionnaires, how to deploy the accelerometers, how to take anthropometric measurements, and how to engage with the participants. The final day of the training was allocated to mock questionnaire administration and enumerators were shadowed by the Field Coordinators. On the field, each enumerator was allocated to two households (four individuals). The coordination of the teams on the field was facilitated by an inclusive open channel of real-time communication.

### Survey data

A series of actions were taken to strengthen the quality of the data collected with the surveys. Surveys were electronically administered with tablets using SurveyCTO (www.surveycto.com), an online platform that allows for the design, collection and monitoring of multiple surveys using Open Data Kit (ODK) format. Electronic data collection uses logical checks on data entry at the time of survey, minimising the risks of data entry mistakes. The almost real-time upload of the questionnaires allowed an initial screening by a Survey Manager of the quality of the data and in rare cases inconsistencies were rectified with an additional visit of the enumerator to the households. The questionnaires were translated into the local language in non-English speaking field sites (Nepali in Nepal and Telugu in India). The ODK Survey definition files have also been archived.

Daily time use and food intakes data were collected through adaptations of validated questionnaires in the literature. We used the time-use questionnaires at hour-intervals adapted from the time-use module in the Women’s Empowerment in Agriculture Index (WEAI)^18^. Through individual face-to-face interview administered by an enumerator, participants recalled how they spent the previous 24-hour time period in one-hour increments^19^. The narrative nature of the interview prompts participants to recall activities around the common parts of a day (sunrise, meals, sunset, praying time) in longer periods of time (1 hour) versus shorter ones (15 minutes), both of which help to mitigate recall errors.

Food intakes were collected though 24-hour dietary recall^20^. Enumerators used structured interviews to ask the participant to recall the type and amount of all food and beverages consumed in the previous 24-hour period. To increase the accuracy of the estimated portion sizes, where culturally acceptable (India and Nepal), we provided bowls and plates of standard dimensions to the respondents.

Despite the protocols in place to mitigate measurement errors, data on time-use and food intakes remain prone to biases inherent in self-reported, recall-based data. In particular, food intake data are recognised to be subject to under-reporting bias which may vary by gender and by anthropometric characteristics^21^. In our study it is possible that the under-reporting of food intakes may have been larger for men than for women – particularly in relation to food eaten outside the home and calories derived from alcohol consumption.

### Accelerometry data

#### Validation of actigraph devices

Actigraph is a medical-grade accelerometer device approved by the U.S. Food and Drug Administration. Its reliability and validity have been extensively assessed^22,23^ and these devices have been used in multiple studies involving free-living humans in various settings^24–26^. Three axes Actigraph devices have been validated against the golden standard (Doubly Water Labels)^27,28^. ActiGraph devices are also being used in large scale collection of physical activity data, e.g. UK’s National Diet and Nutrition Survey (NDNS), USA’s National Health and Nutrition Examination Survey (NHANES), and USA’s Women’s Health Study. Recently, there has been an increased use of Actigraph devices in low- and middle-income countries settings^11,12,29–32^.

#### Failure of devices

In two instances (0.4%) the devices failed, and it was not possible to retrieve the data (Week 2 and 4 in Ghana, female participant). In one case, the battery ran out on the 6^th^ day, but the data recorded until then was successfully retrieved (Week 2 in Ghana, male participant).

#### Device wear compliance

Participants’ compliance in wearing accelerometers devices is key in physical activity studies. Compliance rate based on the number of hours during waking hours (4 am–11 pm in Ghana and Nepal and 5 am–11 pm in India) of non-wear time shows an 81–94% full compliance rate and a 94–97% of days with a non-wear time less than three hours (Table 5). The non-wearing time does not take into account the non-wearing time during the day that was associated with ‘sleeping’ or ‘bathing’ activities reported in the time use data. In these cases, the non-wearing time was consistent with the participant’s activities. Qualitative findings suggest the most common cause of non-wearing was participation in social events (funerals and festivals) when participants did not feel comfortable wearing the devices in public. Compliance rates are significantly higher than studies in Europe/USA^33^ and other low- and middle-income countries^29,32^.

**Table 5:** Device wear compliance.

Missing hours/day	Ghana		India		Nepal	
	N	%	N	%	N	%
0	1,040	94.12	915	81.7	920	85.34
1	34	3.08	90	8.04	93	8.63
2	5	0.45	47	4.2	24	2.23
3	8	0.72	33	2.95	9	0.83
4	6	0.54	6	0.54	8	0.74
5	1	0.09	6	0.54	4	0.37
6	3	0.27	3	0.27	3	0.28
7	2	0.18	3	0.27	4	0.37
8	0	0	3	0.27	5	0.46
9	2	0.18	5	0.45	1	0.09
10	2	0.18	6	0.54	1	0.09
11	1	0.09	2	0.18	2	0.19
12	1	0.09	1	0.09	2	0.19
13	0	0	0	0	1	0.09
14	0	0	0	0	1	0.09
TOTAL	1,105	100	1,120	100	1,078	100

Note: The table does not include the two accelerometer devices that failed (14 days lost) and one case in which the battery extinguished before the end of the data collection (1 day lost).

Two key features embedded in our study design contributed to the high compliance rates. First, the daily visits of the enumerators to administer the individual questionnaires provided an opportunity to check compliance on the field, and where required, to remind the participants to wear the devices. Secondly, the long time during which the participants were enrolled in the study allowed them to get used to wearing the devices in the morning. In fact, most of the non-wear time is during the first week or during the first day of the week. In addition, before beginning the study we ran a series of meetings with the communities selected to provide all the information needed regarding accelerometer devices (potential participants had the opportunity to try out the devices) and to build trust between the survey team and the participants. This proved to be key for the overall completeness and quality of the data.

#### Steps to avoid mix-up of devices between respondents in a household

We put in place multiple strategies to mitigate the risk of involuntary exchange of accelerometer devices between respondents within a household. In addition to instructing the participants about the issue, a visible identification feature (a pattern sewn into the belt or a pin) were included in the belt to identify the device to be worn by the male and female respondent in a household. The daily questionnaire included a series of questions for enumerators to check the participant was wearing the assigned device without having to check the serial numbers.

### Outliers

Table 6 reports the outliers in the accelerometer data and food intake. Outliers have been defined as data points that are more than 1.5 interquartile range above the third quartile or below the first quartile.

**Table 6:** Outliers.

	Accelerometer		Food intake	
	N	%	N	%
Ghana	26	2.4	0	0
India	37	3.3	41	3.7
Nepal	20	1.9	19	1.7

## Usage Notes

Datasets can be used as stand-alone, yet richer analysis can be done linking the various sources of information. The datasets can be linked across the different levels (hour, day, week, individual, households) matching key variables that identify the level of analysis (Table 7).

**Table 7:** Key variables identifying the level of analysis.

Country	Household ID (HH)	Individual ID (IND)	Week (WEEK)	Day (DAY)
Ghana - GH (1) India - IN (2) Nepal – NP (3)	Unique number identifying the households at country level (0–30)	Male - M (1) Female – F (2)	Land preparation (1) Seeding and sowing (2) Land maintenance (3) Harvest (4)	Day 1 (1) Day 2 (2) Day 3 (3) Day 4 (4) Day 5 (5) Day 6 (6) Day 7 (7)

Accelerometery data can be analysed with Actilife^TM^ software (www.actigraphcorp.com/actilife/), R (e.g. using the package ‘Actigraphy’ or ‘accelerometry’), or Matlab. For example, using Freedson VM3 combination formula^23^ energy expenditure can be computed as follow: Kcals/min = 0.001064 × VM + 0.087512 (BM) − 5.500229 or Kcals/min = CPM × 0.0000191 × BM (if VMCPM < 2453), where VM = Vector Magnitudine = $\sqrt{{(axis\_1)}^{2}+{(axis\_2)}^{2}+{(axis\_3)}^{2}}$; VMCPM = Vector Magnitude Counts per Minute; CPM = Counts per Minute; and BM = Body Mass in kilograms. Thresholds on CMP can also provide physical activity levels: light (0–2690 CPM), moderate (2691–6166 CPM), vigorous (6167–9642 CPM), and very vigorous (9643–∞ CPM)^23^.

The supplementary material deposited includes a translation of the activities from the local language to English. Food intakes can be converted into macro- and micro-nutrients using Food Composition Tables (FCTs). FCTs including local dishes are available for Ghana^34^, Nepal^35^, and India^36^. Nonetheless, there is the possibility that the crop varieties grown and consumed in the study areas may not correspond with the exact variety reported in the FCTs. This is more likely to affect the conversion to micro-nutrients, with calories and macro-nutrients less prone to differences across varieties of the same species^37^.

## Data Availability

The data were exported from SurveyCTO in Stata format. The data summarizing and files’ renaming were performed with Stata 16. The code used can be provided upon request.
